# OntoQuery: easy-to-use web-based OWL querying

**DOI:** 10.1093/bioinformatics/btt514

**Published:** 2013-09-05

**Authors:** Ilinca Tudose, Janna Hastings, Venkatesh Muthukrishnan, Gareth Owen, Steve Turner, Adriano Dekker, Namrata Kale, Marcus Ennis, Christoph Steinbeck

**Affiliations:** ^1^Cheminformatics and Metabolism, European Bioinformatics Institute, Cambridge, UK, ^2^Swiss Center for Affective Sciences, University of Geneva, Geneva, Switzerland and ^3^Evolutionary Bioinformatics, Swiss Institute of Bioinformatics, Lausanne, Romandy, Switzerland

## Abstract

**Summary:** The Web Ontology Language (OWL) provides a sophisticated language for building complex domain ontologies and is widely used in bio-ontologies such as the Gene Ontology. The Protégé-OWL ontology editing tool provides a query facility that allows composition and execution of queries with the human-readable Manchester OWL syntax, with syntax checking and entity label lookup. No equivalent query facility such as the Protégé Description Logics (DL) query yet exists in web form. However, many users interact with bio-ontologies such as chemical entities of biological interest and the Gene Ontology using their online Web sites, within which DL-based querying functionality is not available. To address this gap, we introduce the OntoQuery web-based query utility.

**Availability and implementation:** The source code for this implementation together with instructions for installation is available at http://github.com/IlincaTudose/OntoQuery. OntoQuery software is fully compatible with all OWL-based ontologies and is available for download (CC-0 license). The ChEBI installation, ChEBI OntoQuery, is available at http://www.ebi.ac.uk/chebi/tools/ontoquery.

**Contact**: hastings@ebi.ac.uk

## 1 INTRODUCTION AND MOTIVATION

Ontologies are being developed throughout the life sciences to enable standardization of annotation, sophisticated database querying and information visualization (Lambrix, 2004; Schuurman and Leszczynski, 2008). The Gene Ontology (GO; The Gene Ontology Consortium, 2000) was one of the earliest such ontologies developed for the standardization and aggregation of annotations about gene product functions across a wide number of biological databases. Another widely used ontology is the Chemical Entities of Biological Interest (ChEBI) ontology (Hastings *et al.*, 2013), which serves as a reference for chemical entities and their biological activities.

For many bio-ontologies, a web-based browsing interface (such as amigo.geneontology.org for the GO) allows searching based on text strings and other associated metadata. Some basic searching based on the structure of the ontology may be available. For example, the ChEBI advanced search provides searches based on pre-indexed expanded relationship paths (de Matos *et al.*, 2010). Programmatic access is provided via libraries such as the Web Ontology Language (OWL) Application Programming Interface (API) library in Java (Horridge and Bechhofer, 2009). Furthermore, various desktop tools provide interfaces that allow browsing and querying of ontologies and associated knowledge bases, most prominent of which is the Protégé ontology editor (http://protege.stanford.edu/).

Protégé provides a query utility (called ‘DL query’) that enables sophisticated logical interrogation of the ontology using the accessible Manchester syntax (Horridge and Patel-Schneider, 2009). For example, the following query has nested subqueries:

(‘chemical entity’ and has_role

some (insecticide or acaricide))

and has_role some fungicide

This type of ontology query, constructed with labels and nested logical subunits, is not at present available via any web-based ontology interface. The WebProtégé tool, which offers other Protégé functionality online, does not offer DL querying. Query tools including SMART (Battista *et al.*, 2007) do not offer label lookups. Other prominent query languages such as SPARQL-DL require the user to be familiar with the underlying Resource Description Framework (RDF) graph.

It is to address this gap that we have developed the OntoQuery utility, an easy-to-use web-based OWL query facility with label replacement, syntax highlighting and checking and auto-complete.

## 2 TOOL FEATURES

The interface provides syntax highlighting similar to that provided by the Protégé DL query tool. However, unlike Protégé, OntoQuery highlighting distinguishes between classes and properties. As the user types, the system pops up a box with suggestions appropriate to the syntactic position within the query. For example, if a class is expected, as the user types the class name, the tool will look up the labels of classes in the loaded ontology and suggest completion options. However, if the previous term in the query was a class name, the tool will suggest connectives instead (e.g. and). The search matches both at the beginning and in the middle of the ontology entity labels, but the matches at the beginning of the word are listed first. The matched substring is highlighted in the suggestion box.

**Fig. 1.**
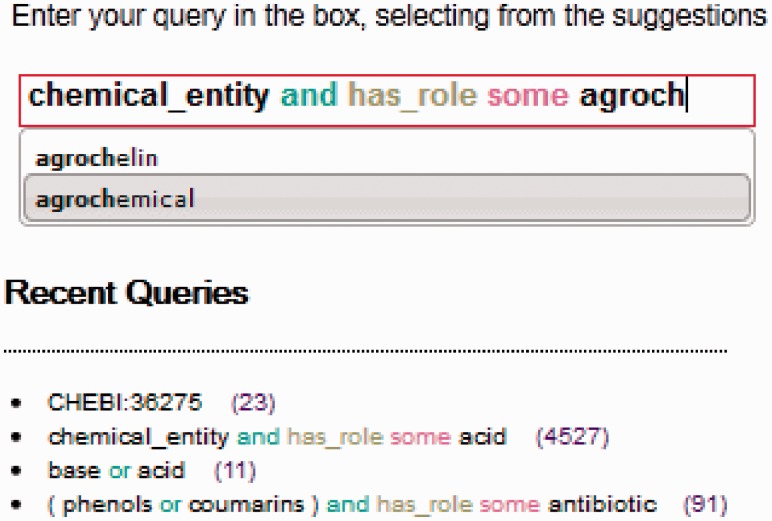
The input box has syntax highlighting and makes suggestions ranking them by relevance and highlighting the matches in the suggestions list. The recent queries indicate the validity of the syntax and the number of results

A recent queries box is also part of the graphical user interface, where at most 10 historical queries are listed with information about the number of results and whether the query was valid or not. Clicking on a historical query loads the query text into the query box, where it can be edited or re-executed easily. The search box and recent queries facility are illustrated in Figure 1.

The OWL language constructs that are available to the auto-suggestion facility are configurable as part of the installation process. Cardinality restrictions are not suggested in the ChEBI installation because ChEBI does not use them, so providing the option to a user could be confusing. However, as the expressivity of the ontology increases, it is a simple matter to extend the expressivity of the query tool in the settings.

The queries will return all descendents (not just direct subclasses) matching the logical definition expressed in Manchester syntax. The use of OWL reasoning means that queries are answered based on an open world assumption. This means that anything not explicitly stated is not available to the reasoner. In particular, queries using ‘not’ only return results explicitly known to be disjoint from the query term because a disjoint axiom is included. ChEBI’s use of disjoint axioms is described in Hastings *et al.* (2013).

## 3 IMPLEMENTATION

The implementation is based on two existing APIs: the OWLTools API (http://code.google.com/p/owltools/) and the JFact API (http://sourceforge.net/projects/jfact/). On the server side, the ontology is loaded, inferences are pre-computed and then query functionality is exposed to the client via a web service. Performance is comparable with query execution in the Protégé tool, once loading and reasoning have been completed. The ontology may be reloaded via an administrator URL as needed.

Queries are sent to the server for syntax checks while they are being typed as well as when the query is submitted. The translation of labels to IDs and the parsing of the query to OWL Manchester syntax are performed on the server. Parsing errors are translated into a user-friendly informative format. The client-side JavaScript is responsible for most of the functionality of the input box, i.e. determining the possible type of the next token for suggestion, ranking of the suggestions’ relevance and syntax coloring. However, it is the server that returns fixed length lists of class names matching the introduced pattern for auto-suggestion. For the syntax-aware auto suggestion, we have implemented our own simple automaton, and for the ranking of suggestions, we use a custom-made metric. We use the Levenshtein distance and the position of the first match, assigning a bigger weight to the match position. Usability testing was conducted using ChEBI curators, who are trained chemists and not software or logic experts.

Although the tool was initially developed for ChEBI, it is applicable to any ontology. Installation for another ontology requires specifying the online ontology file and setting a few installation variables. A default result view lays out the results with IDs, labels and the term Uniform Resource Identifier (URI) as the hyperlink.

The tool has been tested on Firefox version 22 and on Google Chrome version 28.

## 4 LIMITATIONS AND FUTURE WORK

Although both labels and IDs can be used in queries, only labels are available in the auto-suggest facility at present. Only the structure of the ontology is available for querying, and additional associated metadata other than labels is not yet available. While Protégé delimits multi-word labels by enclosing them in single quotes, motivated by the complexity of chemical names in ChEBI where some labels may contain quote characters, OntoQuery has used underscores to replace spaces in multi-word labels. Furthermore, parentheses used for grouping need to be separated by spaces, as labels may contain parenthesis characters. Future work will involve making these aspects configurable on a per-ontology basis, exposing more metadata from annotations to the querying, and making the suggestions for autocomplete aware of the range and domain of properties. We also aim to introduce a download facility for the results, and allow for ontology query types other than strict descendents, e.g. ancestors.

*Funding*: This work has been supported by the BBSRC, grant agreement number [BB/G022747/1] within the ‘Bioinformatics and biological resources’ fund.

*Conflict of Interest*: none declared.
